# Intestinal parasites prevalence and related factors in school children, a western city sample-Turkey

**DOI:** 10.1186/1471-2458-4-64

**Published:** 2004-12-22

**Authors:** Pinar Okyay, Sema Ertug, Berna Gultekin, Ozlem Onen, Erdal Beser

**Affiliations:** 1 Department of Public Health, Adnan Menderes University Medical Faculty, Aydin, Turkey; 2 Department of Microbiology and Clinical Microbiology, Adnan Menderes University Medical Faculty, Aydin, Turkey; 3 Department of Microbiology and Clinical Microbiology, Adnan Menderes University Medical Faculty, Aydin, Turkey; 4 Department of Public Health, Adnan Menderes University Medical Faculty, Aydin, Turkey; 5 Department of Public Health, Adnan Menderes University Medical Faculty, Aydin, Turkey

## Abstract

**Background:**

Intestinal parasitic infections are amongst the most common infections worldwide. Epidemiological research carried out in different countries has shown that the social and economical situation of the individuals is an important cause in the prevalence of intestinal parasites. Previous studies in Turkey revealed a high prevalence of intestinal parasitic infection. The objectives of the current study were to determine the prevalence of intestinal parasitic infections in Aydin among 7–14 years old school children and to identify associated socio-demographic and environmental factors, behavioral habits and also related complaints.

**Methods:**

Multistage sampling was used in the selection of the study sample. A questionnaire, cellulose adhesive and a stool specimen examination were done.

**Results:**

A total of 456 stool specimens were collected. 145 students (31.8%) were infected with one or more intestinal parasites. 29 (6.4%) of the students were infected more than one parasite, 26 (5.7%) with two parasites and 3 (0.7%) with three parasites. The three most common were *E. vermicularis*, *G. intestinalis *and *E. coli*. Intestinal parasite prevalence was higher in rural area, in children with less than primary school educated mother, in children who use hands for washing anal area after defecation, and in children who use toilet paper sometimes or never. The relation between child health and mother education is well known. Children were traditionally taught to wash anal area by hand. Toiler paper usage was not common and might be due to low income or just a behavioral habit also. Most of the complaints of the study population were not significantly related with the intestinal parasitic infection.

**Conclusions:**

Intestinal parasitic infection is an important public health problem in Aydin, Turkey. Rural residence, mother education less than primary school, sometimes or never usage of toilet paper, and washing anal area by hands after defecation were the significant associations. Interventions including health education on personal hygiene to the students and to the parents, especially to mothers are required. The ratio of uneducated women should be declined with specific programs. A multisectoral approach is needed.

## Background

Intestinal parasitic infections are amongst the most common infections worldwide. It is estimated that some 3.5 billion people are affected, and that 450 million are ill as a result of these infections, the majority being children. These infections are regarded as serious public health problem, as they cause iron deficiency anemia, growth retardation in children and other physical and mental health problems [1].

Epidemiological research carried out in different countries has shown that the social and economical situation of the individuals is an important cause in the prevalence of intestinal parasites. In addition, poor sanitary and environmental conditions are known to be relevant in the propagations of these infectious agents [2-4].

Previous studies at various institutions in Turkey revealed a high prevalence of intestinal parasitic infections among the following populations: 6–16 year olds (10.8%), 12–16 year olds (48.0%), 7–15 year olds (55.1%), and 6–12 year olds (88.0%) [5-8]. However, almost all of the studies were performed in isolated groups, such as collecting all samples from children attending the same school. Furthermore the majority of the studies were performed in the eastern part of Turkey. They have limitations in regards to giving an idea about all age groups in the region. Although there are studies on interactions between infection and socio-demographic, environmental factors, and behavioral habits from eastern regions, to our knowledge, there is lack of adequate information for the western part of Turkey [7,9].

The objectives of this current study were to determine the prevalence of intestinal parasitic infections in Aydin among 7–14 year old school children and its relation to socio-demographic factors, environmental factors, behavioral habits and complaints related to intestinal infections.

## Methods

### Study population

The data for the present study was acquired from the primary schools in urban and rural areas of Aydin, a city in the western part of Turkey. The study design was cross-sectional.

The sample size was calculated on a prevalence of 30%, d = 0.05 at a confidence level of 95%. A design effect of 2 was used to allow for multistage sampling [10]. The calculated study population size was 639. Multistage sampling was used in the selection of the study sample. Aydin was separated into four regions according to the socio-economic and health data taken from Directorate of Health. From these regions two schools were selected randomly, one from the urban communities and one from rural. The children were selected from the classes randomly based on the age, gender and weight for the population. First to eighth graders were included in the study, and the total numbers of the students enrolled in the classes were 74, 60, 70, 72, 72, 107, 104, and 81 students, respectively. Permission was obtained from Directorate of Education.

### The questionnaire and family information form

The questionnaire contained four sections: 1. Socio-demographic data: age, gender, residence, education and occupation of parents, number of adults and children in the family and birth order of the child; 2. Environmental factors: housing conditions (ownership of the house, number of rooms and bathrooms) and water supply; 3. Behavior habits: type of toilet commonly used, hand washing (no washing/washing with only water/washing with soap), washing anal area by hands after defecation (yes/no), usage of toilet paper (always/sometimes/never); 4. Complaints: abdominal pain, nausea/vomiting, lack of appetite, abdominal distention, intestinal dismotility, salivation during sleeping, headache, irritability in sleeping, perianal itching, teeth grinding, and history of parasitic infections.

An informational document about the study, including how to supply a stool specimen and a cellulose tape slide, was given to each participant for their family.

### Intestinal parasitic examination

Mothers were asked to perform one cellulose tape test on their child who was participating in the study. Laboratory slides were provided with cellulose tape attached to them. The mother collected material for examination in the early morning prior to bathing or defecation. On the same morning a field worker collected these slides to be microscopically examined.

The stool specimens (0.5–1.5 gr) were collected in labeled plastic vials without preservatives and transported to the laboratory within four hours after collection. They were examined for the presence of parasites by direct wet mount, Lugol's iodine solution and modified formaline-ethyl acetate sedimentation techniques.

The presence of parasites was confirmed when observed by any of the methods above.

### Statistical analysis

A computer program was used for data analysis. The descriptive data was given as a mean ± standard deviation (SD). The chi-squared test and Student t-test were used for the analytic assessment. The differences were considered to be statistically significant when the p-value obtained was less than 0.05.

## Results

A total of 456 (71.4%) samples for both stool specimens and cellulose tape slides were collected. The response rate to the questionnaire was lower with 367 (57.4 %). The mean age was 10.34 ± 2.27, 10.17 ± 2.30 for girls and 10.51 ± 2.23 for boys. Important socio-demographic characteristics, housing conditions and hygienic habits of children are given in Table 1.

**Table 1:** Important socio-demographic characteristics, housing conditions and hygienic habits of children

Characteristics		No	%
Socio-demographic characteristics			

Residence	Urban	258	56.6
	Rural	198	43.4
Gender	Female	232	50.9
	Male	224	49.1
Education of mother	No education/primary school incomplete	68	19.0
	Primary/secondary school	269	75.4
	High school and more	20	5.6
Education of father	No education/primary school incomplete	21	5.8
	Primary/secondary school	292	81.4
	High school and more	46	12.8

Housing conditions			

	Owner	239	66.0
	3 rooms and less	154	43.5
	4 rooms and more	200	56.5
	1 toilet	180	49.9
	2 and more toilet	181	50.1
	5 and less people living in	267	74.4
	6 and more people living in	92	25.6
	Municipal water network	232	68.6

Hygienic habits			

Type of toilet commonly used	Modern style	86	26.5
	Traditional style	135	41.5
	Both	104	32.0
Toilet paper	Always	206	57.1
	Sometimes	105	29.1
	Never	50	13.9
Washing anal area by hands after defecation	Yes	138	39.4
	No	212	60.6
Washing hands with soap after toilet	Always	308	85.3
	Sometimes	51	14.1
	Never	2	0.6
Taking a bath	Once a day	18	5.2
	Three times a week	161	46.1
	Once a week or less	170	48.7

In all, 145 students (31.8%) were infected with one or more intestinal parasites. 29 (6.4%) of the students were infected with more than one parasite, 26 (5.7%) with two parasites and 3 (0.7%) with three parasites. The most common was *Enterobius vermicularis (E. vermicularis*) with 63 (13.8%) pure and 20 (4.4%) with multiple infections, in total 83 (18.2%) infected children. The second was *Giardia intestinalis *(*G. intestinalis*) with 28 (6.1%) pure and 21 (4.6%) with multiple infections, in total 49 (10.7%) infected children. The third one was *Entamoeba coli *(*E. coli*) with 21(4.6%) pure and 15 (3.3%) multiple infections, in total 36 (7.9%) infected children. *G. intestinalis *was the most commonly found parasite in multiple infections. The distribution of parasites is given in Table 2.

**Table 2:** The parasites distribution of the study population

Parasites	No.	%
Single		
E. vermicularis	63	13.8
G. intestinalis	28	6.1
E. coli	21	4.6
H. nana	4	0.9
Total	116	25.4
		
Multiple		
E. vermicularis+G. intestinalis	11	2.4
E. vermicularis+E. coli	8	1.8
G. intestinalis+E. coli	4	0.9
G. intestinalis+H. nana	2	0.4
G. intestinalis+Taenia spp.	1	0.2
G. intestinalis+E. coli+H.nana	2	0.4
E. vermicularis+G. intestinalis+E. coli	1	0.2
Total	29	6.4
		
Overall total	145	31.8

No statistically significant difference was observed between presence of intestinal parasites and gender, (p = 0.805), and also age (p = 0.916). The prevalence of intestinal parasites were significantly higher (p = 0.042) in the rural area (36.9%) than in the urban area (27.9%). A summary of significant relations observed in overall intestinal parasitic infections for the study population are given in Table 3.

**Table 3:** Significant relations for the intestinal parasitic infection in the study population

Risk Factor	Overall infection		χ^2^	p
			
	n	%		
Residence				
Urban	72	27.9	4.415	0.042
Rural	73	36.9		
Mother education				
Less than primary school	29	42.6	4.436	0.035
Primary school and more	85	29.4		
Toilet paper				
Always	50	24.3	13.596	0.000
Sometimes/never	66	42.6		
Washing anal area by hands after defecation				
Yes	55	39.9	5.503	0.019
No	59	27.8		

Parasite-specific significant relations were the following: The prevalence of E. coli infections was significantly higher (p = 0.010) in the rural area (11.1%) than in the urban area (5.0%). Mother education less than a primary school education (p = 0.012), washing anal area by hands after defecation (p = 0.013) were the significant relations for *G. intestinalis*, where sometimes or non-usage of toilet paper was significant for *G. intestinalis *(p = 0.008) and for *E. vermicularis *(p = 0.024) both. Family size was larger in the group infected with *G. intestinalis *(p = 0.029). The prevalence of intestinal parasitic infection was 31.0% in the group using a municipal water network and 34% in the group lacking a municipal system (p = 0.592). The prevalences of *G. intestinalis *in these two groups were 9.9% and 16.0% (p = 0.106) respectively. No further significant relationships were found between intestinal parasitic infection and environmental or behavioral factors.

The most frequent complaint related with any parasite infection was intestinal dismotility (40.0%). Nausea/vomiting (37.7%) was second and abdominal distention (37.1%) was third. All of the complaints were seen in higher prevalence for *E. vermicularis *infections than *G. intestinalis *and *E. coli*.

## Discussion

It was found that approximately one-third (31.8%) of the students, ages 7 to 14, in Aydin were infected by intestinal parasites. In a sample within the same age group in Izmir, a city also in the western part of Turkey, the prevalence for intestinal parasites was 22.4%, with *E. vermicularis *(16.0%) and *G. intestinalis *(11.9%) being the two most common infections, as was observed in Aydin [11]. In another study performed in Izmir, the prevalence of infection was 45.3% for *E. vermicularis*, 21% for *G. intestinalis*, 10% for *H. nana*, 4.3% for *E. coli*, 0.03% for *Ascaris lumbricoides *(*A. lumbricoides*), and 0.03% for *Trichuris trichiura *(*T. trichiura*). No *Taenia *was found [12]. In this current study; the most frequently observed parasites were *E. vermicularis*, *G. intestinalis*, and *E. coli*, 18.2%, 10.7% and 7.9%, respectively. Higher prevalence was found in the studies from the eastern part of Turkey, where the socio-economic and environmental conditions were lower. Additionally, it was observed that the types of parasites found in this study where different than those found in the eastern part of Turkey. A survey conducted among 1001 children in four elementary schools in Sanliurfa found parasites in 88% of the stool samples examined (50% *A. lumbricoides*, 53% *T. trichiura*, 22% *G. intestinalis*, and 11% *E. coli*); unfortunately, no data on *E. vermicularis *was given, because samples with cellulose tape slides were not taken [7]. In an another study from the eastern region, in an elementary school age group, 48.12% *A. lumbricoides*, 4.43% *T. trichiura *and 15.35% *G. intestinalis *were found [13]. In the last study, samples with cellulose tape slides were not taken.

Geohelmint infections (*Ascaris*, *Strongyloides *and *Trichuris*) were of lower prevalence in the western part of Turkey, but of higher prevalence in the eastern part. This difference may be due to improper toilet facilities which require individuals to defecate in areas around their homes as well as the use of fecal material for fertilizer in gardens in the eastern parts of Turkey [13-15]. In a study from the eastern region, 42.2% of children were found to be working in gardens watered with contaminated sewage and eating the vegetables of those gardens. There were 44.7% *A. lumbricoides*, 11.7% *T. trichiura *infections in these children while there was 12.2 %*A. lumbricoides *and 6.6% *T. trichiura *infections in the control groups [14].

Enterobiasis occurs worldwide, usually involving school-aged children [16]. In general *E. vermicularis *infection is transmitted by hand to mouth and/or person to person directly. High prevalence of *E. vermicularis *in the current study might be due to improper hygiene including not washing hands with soap after defecation, before eating and preparing foods. In the study area, there are two traditional methods of cleaning anal areas after defecation: (1) washing the anal area by hand with tap water (2) a piece of cloth is used to clean the anal area after defecation. The cloth is used multiple times until the person decides that it has become too dirty after which it is washed and reused. These improper cleaning practices after defecation could be the probable causes behind autoinfection. The higher prevalence of *E. vermicularis *could also be explained by the highly infectious nature of the parasites.

*G. intestinalis *and *E. coli *were the most common intestinal protozoa among the study population. Both can be transmitted orally by drinking infected water and both are environmental contaminants of the water supply. The water supply is really an important risk factor for the Giardiasis, and several large outbreaks of giardiasis have resulted from the contamination of municipal water supplies with human waste [17]. The ingestion of contaminated water is a common problem in Turkey countrywide due to the lower quality of water and faulty sewage lines. The problem is greater in the rural areas that do not have a municipal water network or sewage system [18]. Contamination of drinking water with *Giardia spp*. has been increasingly recognized over the past 10 years as a cause of water-borne diseases in humans [19]. *Giardia *cysts have been isolated from water supplies in different parts of the world [20,21]. Epidemic giardiasis may be related to drinking water [22]. *G. intestinalis *and *E. coli *are most common in the western part of Turkey [11]. In a study assessing giardiasis cases in Turkey published within the last 15 years, the prevalence of *G. intestinalis *was found 11.6% in the western part of Turkey [23]. From the study, 68.6% of the study group uses municipal water, while the others either use a chlorinated collection tank with a crude water network or purchase bottled water. Although no statistically significant difference was observed, the prevalence of *G. intestinalis *was lower in the group using municipal water (9.9%) than the other group (16.0%) in the current study. It is thought that an in-depth assessment should be done on the ways that drinking water becomes contaminated.

In this current study, there were no cases of *Taenia spp*. Since the consumption of pork and pork products are forbidden for Muslims this may account for the absence of *T. solium *cases in the population. It is also a common practice to eat uncooked meat in the eastern part of Turkey, but not in the western part. *T. saginata *infections are observed in the eastern studies, with prevalence of 13.8% and 12.9% [19,24]. However, in the western region infections by this parasite were not observed in this or in another previous study [12].

Differences due to gender were not observed in this current study. These results were similar to a study conducted in the central part of Turkey [24]. In this current study; the prevalence of intestinal parasitic infection was higher in the rural areas. A similar result was found in the central region of Turkey where the prevalence of intestinal parasites was higher in the rural area [25].

In this current study, most of the complaints by the study population were not significantly related with the intestinal parasitic infection. For example, perianal itching was noted in 15.8 % of the study population. There was no significant difference in the prevalence of this symptom in pinworm infected and non-infected children. Furthermore, no association was found between the prevalence of pinworm infection and a history of teeth grinding, colic, enuresis, and irritability. The complaints may not have been assessed effectively through the use of only a questionnaire without an interview, or they might not be useful for every individual's diagnosis.

The prevalence of intestinal parasites was higher in groups where the mother in the household had less than a primary school education, where the hand is habitually used for the cleaning of the anal area and where toilet paper is seldom or never used. The relation between a child's health and the mother's education is well known. Health indicators of children whose mother's education level is lower are always worse [26]. In the last two groups, the habits of the children are a factor, along with a cultural dimension. They were taught to clean the anal area by washing with the hand. Toiler paper usage was not common, possibly due to low income. Usage of a piece of cloth instead of toilet paper was also common.

The major limitation of the current study was a low response rate. The assessment would have been more valuable if a higher response rate could have been obtained. But, it was thought that the results were still important because there is little knowledge on the data of the region. Additionally, the current study was the first population-based study for the region. Although an important number of risk factors were discussed, a few risk factors (e.g.: shoe wearing) were not evaluated in the current study. This might have been another limitation of the study. The important risk factors for the region were evaluated in the study.

## Conclusion

As a conclusion, intestinal parasitic infection is an important public health problem in Aydin, Turkey. Rural residence, households where the mother has less than a primary school education, periodic or non-usage of toilet paper, and the washing of the anal area by hand after defecation were the significant associations. Interventions including health education on personal hygiene to the students and to the parents, especially to mothers are required. The ratio of uneducated women should decline with specific programs. A multisectoral approach is needed.

## Competing interests

The author(s) declare that they have no competing interests.

## Authors' contributions

PO planned the research, performed the sampling and statistical analyzes and wrote draft and final version of the manuscript. SE contributed in planning the research, performed parasitic examinations and contributed discussing the results and writing manuscript. BG performed parasitic examinations and contributed discussing the results. OO organized the work in the schools and participated in its design. EB participated in initial study design, coordinated the study and revised the manuscript. All authors read and approved the final manuscript.

## Pre-publication history

The pre-publication history for this paper can be accessed here:


